# Repetitive transcranial magnetic stimulation improves cognitive function of Alzheimer's disease patients

**DOI:** 10.18632/oncotarget.13060

**Published:** 2016-11-03

**Authors:** Junwu Zhao, Zhenguang Li, Yannan Cong, Jinbiao Zhang, Ming Tan, Haixia Zhang, Na Geng, Mengfan Li, Wenwen Yu, Peiyan Shan

**Affiliations:** ^1^ Department of Neurology, Qilu Hospital of Shandong University, Jinan, Shandong, China; ^2^ Department of Neurology, Weihai Municipal Hospital, Weihai, Shandong, China; ^3^ Department of Clinical Medicine (Neurology), Weifang Medical University, Weifang, Shandong, China

**Keywords:** rTMS, cognitive function, AD

## Abstract

Repetitive transcranial magnetic stimulation (rTMS) acts as a kind of widely-applied and non-invasive method in the intervention of some neurological disorders. This prospective, randomized, double-blind, placebo-controlled trial investigates the effect of rTMS on 30 cases of Alzheimer’s disease (AD) participants, who were classified into mild and moderate groups. Neuropsychological tests were carried out using the AD Assessment Scale-cognitive subscale (ADAS-cog), Mini-Mental State Examination (MMSE), Montreal Cognitive Assessment (MoCA), and World Health Organization University of California-Los Angeles, Auditory Verbal Learning Test (WHO-UCLA AVLT) before, immediately after, and 6 weeks after the intervention. In this work, data from 30 AD patients revealed that there was no obvious interaction effect of time-by-group. The ADAS-cog, MMSE and WHO-UCLA AVLT score in the rTMS group was significantly improved compared with baselines at 6 weeks after treatment (all *p*<0.05). Meanwhile, MoCA scores were also obviously ameliorated in the mild AD patients with rTMS. Besides, subgroup analysis showed that the effect of rTMS on the memory and language of mild AD patients was superior to those of moderate AD patients. In conclusion, our findings suggested that repetitive transcranial magnetic stimulation improves cognitive function, memory and language level of AD patients, especially in the mild stage of AD. Thus, rTMS can be recommended as a promising adjuvant therapy combined with cholinesterase inhibitors at the mild stage of AD patients.

## INTRODUCTION

Alzheimer's disease acts as the most common one in all kinds of dementia, especially in the elderly populations, and the prevalence of AD is always increasing in recent years [1]. AD was featured as classic cognitive deficits (memory impairment), change of behaviors, insomnia, and dysfunction of body autonomy. The treatment at an early stage is very important. Most of recent treatment options for AD patients mainly concentrated on preventing the decline of cognition by way of medications [2, 3]. In view of less pharmacological options, other nonpharmacological therapeutic strategies were emerging in recent years [4, 5], of which the transcranial magnetic stimulation has been recommended as an useful and effective noninvasive option.

Repetitive transcranial magnetic stimulation has been reported as a non-invasive tool to regulate and balance the activity of neural cells. It should be noted that rTMS has some advantages in the safety and non-invasive property [6, 7], and rTMS can combine some different medications and other stimuli [8–10]. To date, many clinical and basic studies have indicated that rTMS can effectively improve the clinical manifestation of AD, Parkinson's disease (PD) and schizophrenia. Mechanically, rTMS is able to mediate the neural plasticity and reduce an imbalance between excitation and inhibition signals [11–13]. As reported, TMS stimulated cortical neurons by generation of magnetic fields to activate the synaptic activities of neuronal circuits in the central neural system [14]. High-frequency TMS has been used for various psychiatric and neurological diseases, including depression, schizophrenia, and Parkinson's disease [15]. However, the role of rTMS in improving cognitive functions of AD patients was less reported.

This study aims to investigate the clinical efficacy of rTMS in ameliorating cognitive levels of patients with AD by the prospective, randomized, double-blind and placebo-controlled trial, and then we analyzed the clinical efficacy of rTMS in mild and moderate AD patients. Our findings suggested that repetitive transcranial magnetic stimulation improves cognitive level, memory and language of AD patients, especially in the mild stage of AD patients. Thus, rTMS can be recommended as a promising adjuvant therapy combined with cholinesterase inhibitors at the mild stage of AD patients.

## RESULTS

### Baseline level of participants

To figure out the baseline of participants, we firstly analyzed the demographic differences, including age, male-to-female ratio and donepezil level. In the present study, 17 AD patients were enrolled and randomly attributed to the rTMS treatment group and the other 13 participants were also randomly attributed into the non-rTMS sham group. As shown in Table 1, we found that there were no significant differences between the treatment and control groups in age (*p* = 0.313), male-to-female ratio (*p* = 1.000), education level (*p* = 0.921), and donepezil level (*p* = 0.751), indicating that both groups can be used as this scientific trial. During this trial, three participants including two patients in rTMS treatment group and one in the sham group had the adverse effect, and they had the mild headache and fatigue after the first treatment. But these three participants were willing to finish this trial. Subsequently they did not have these adverse effects. Mostly, we did find no significant differences between these two groups in related baseline levels including ADAS-cog (*p* = 0.474), MMSE (*p* = 0.536), MoCA (*p* = 0.810) and WHO-UCLA AVLT scores (0.591) (Table 1).

**Table 1:** Baseline characteristics of all participants

Indications	Mean (*n* = 30)	rTMS group (*n* = 17)	Sham group (*n* = 13)	*p* -value
Age (years)	70.8±5.6	69.3±5.8	71.4±5.2	0.313
Females (%)	56.7	58.8	54.8	1.000
Education level (years)	4.9±2.3	4.8±1.9	4.9±3.5	0.921
Donepezil (mg)	8.2±2.2	8.0±2.5	8.3±2.6	0.751
ADAS-cog	23.6±6.9	22.6±5.9	24.2±6.1	0.474
MMSE	22.5±2.7	22.2±2.8	22.8±2.3	0.536
MoCA	17.9±5.8	17.5±6.2	18.1±7.3	0.810
WHO-UCLA AVLT	33.1±8.7	32.5±7.9	34.1±8.1	0.591

### ADAS-cog score

**Figure 1:**
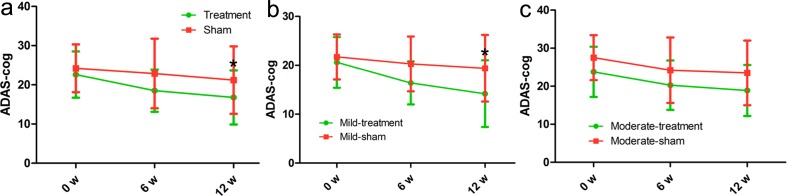
Differences in ADAS-cog score at each time point (baseline, immediately after, 6 weeks after the treatment) There was no significant time-by-group interaction in spite of significant improvements in the treatment group. The red line represents the sham group and the green line represents the treatment group. *p < 0.05, v.s. baseline.

Using ADAS-cog scale, we compared ADAS-cog score in the rTMS treatment group with that in the sham group based on the baselines. Firstly, there was no significant differences in the group-by-time interaction (*p* = 0.332), with which we can figure out the role of the ADAS-cog score in the treatment group and the sham group very well. Next we found that all 17 AD patients in the rTMS group showed an significant increase by 4.1 ADAS-cog scores after the first 6 weeks of rTMS treatment (*p* = 0.042), and a more significant increase by 5.8 ADAS-cog scores after 12 weeks of rTMS treatment (*p* = 0.013). Whereas, the ADAS-cog scores in the sham group exhibited no significant increase by 1.3 and 3.0 points immediately and 6 weeks after non-rTMS treatment (*p* = 0.668, *p* = 0.315, respectively) (Figure 1a, Table 2). Besides, the ADAS-cog score in the mild treatment group significantly improved much more compared with that in the mild sham group (Figure 1b). However, the ADAS-cog score in the moderate treatment group insignificantly improved compared with that in the moderate sham group (Figure 1c), indicating that rTMS has more advantages in treating the mild AD patients.

**Table 2:** Neuropsychological assessment scores at all participants and in the mild and moderate group

Neuropsychological assessments	Group (*n*)	Mean Scores (SD)			*p* value		
		Baseline [B]	Immediate after treatment [6]	6 weeks after treatment [12]	[B] vs. [6]	[B] vs. [12]	Time* group
ADAS-cog	Treatment (17)	22.6 (5.9)	18.5 (5.4)	16.8 (6.9)	0.042*	0.013*	0.332
	Sham (13)	24.2 (6.1)	22.9 (8.9)	21.2 (8.6)	0.668	0.315	
	Mild-treatment (12)	20.6 (5.2)	16.4 (4.4)	14.2 (6.8)	0.044*	0.017*	0.263
	Mild-sham (8)	21.7 (4.6)	20.3 (5.6)	19.4 (6.8)	0.593	0.441	
	Moderate-treatment (5)	23.8 (6.6)	20.3 (6.5)	18.9 (6.7)	0.423	0.278	0.878
	Moderate-sham (5)	27.5 (5.9)	24.2 (8.6)	23.5 (8.5)	0.499	0.413	
MMSE	Treatment (17)	22.2 (2.8)	23.9 (2.5)	25.5 (4.6)	0.071	0.017*	0.557
	Sham (13)	22.8 (2.3)	23.1 (3.3)	24.2 (4.1)	0.790	0.294	
	Mild-treatment (12)	25.6 (2.1)	27.1 (4.1)	29.7 (4.5)	0.147	0.042*	0.639
	Mild-sham (8)	25.8 (2.3)	26.2 (3.5)	28.1 (3.5)	0.791	0.395	
	Moderate-treatment (5)	19.2 (2.5)	20.4 (3.3)	21.7 (4.3)	0.535	0.294	0.812
	Moderate-sham (5)	19.5 (1.9)	20.4 (2.5)	21.5 (2.1)	0.539	0.153	
MoCA	Treatment (17)	17.5 (6.2)	19.8 (6.5)	21.5 (5.9)	0.299	0.063	0.552
	Sham (13)	18.1 (7.3)	19.3 (6.7)	20.1 (6.6)	0.666	0.471	
	Mild-treatment (12)	18.6 (5.1)	21.1 (4.3)	23.1 (5.3)	0.208	0.046*	0.799
	Mild-sham (8)	19.7 (7.5)	20.9 (7.1)	21.4 (7.8)	0.747	0.664	
	Moderate-treatment (5)	16.6 (6.2)	17.9 (5.8)	19.2 (5.5)	0.741	0.503	0.517
	Moderate-sham (5)	17.5 (6.8)	18.4 (5.9)	19.5 (6.6)	0.829	0.650	
WHO-UCLA AVLT	Treatment (17)	32.5 (7.9)	35.8 (7.8)	38.7 (8.9)	0.229	0.039*	0.667
	Sham (13)	34.1 (8.1)	35.8 (7.4)	37.8 (8.7)	0.582	0.273	
	Mild-treatment (12)	35.6 (5.6)	37.9 (6.5)	41.8 (6.6)	0.363	0.021*	0.524
	Mild-sham (8)	35.8 (6.7)	36.6 (6.7)	38.7 (4.5)	0.815	0.327	
	Moderate-treatment (5)	30.5 (7.6)	33.5 (2.3)	35.6 (6.3)	0.423	0.281	0.550
	Moderate-sham (5)	30.6 (6.7)	33.9 (5.4)	36.5 (4.9)	0.416	0.151	

Repetitive measures ANOVA was adjusted with age, gender, education level, and then Bonferroni comparison was used for post-hoc analysis.

*p value<0.05. NA: not applicable.

### MMSE score

**Figure 2:**
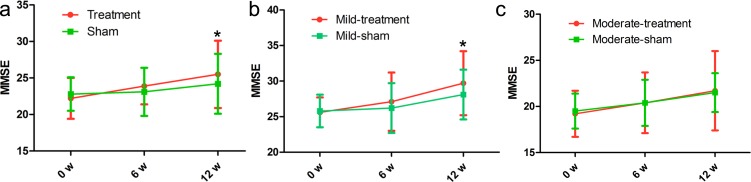
Differences in MMSE score at each time point (baseline, immediately after, 6 weeks after the treatment) There was no significant time-by-group interaction in spite of significant improvements in the treatment group. The red line represents the treatment group and the green line represents the sham group. *p < 0.05, v.s. baseline.

In the treatment group, the mean MMSE score ranged from 22.2 (baseline) to 23.9 (immediately after the treatment) and 25.5 (6 weeks after the end of the treatment), meanwhile, in the sham group, the mean MMSE score ranged from 22.8 (baseline) to 23.1 (immediately after the treatment) and 24.2 (6 weeks after the end of the treatment). According to statistics, there was a significant increase at 6 weeks after the end of the treatment (*p* = 0.017) instead of immediately after the treatment (*p* = 0.071). However, the MMSE score showed no significant change in the sham group (*p* = 0.790, *p* = 0.294, respectively; Figure 2a, Table 2). In addition, the MMSE score in the mild treatment group was significantly improved as compared with that in the mild sham group (Figure 2b). However, the MMSE score in the moderate treatment group was insignificantly improved compared with that in the moderate sham group (Figure 1c), indicating that rTMS has more advantages in treating the mild AD patients.

### MoCA score

**Figure 3:**
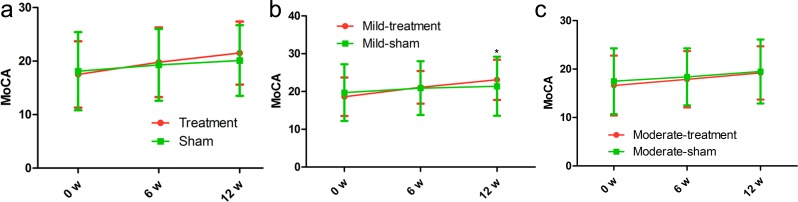
Differences in MoCA score at each time point (baseline, immediately after, 6 weeks after the treatment) There was no significant time-by-group interaction in spite of significant improvements in the treatment group. The red line represents the treatment group and the green line represents the sham group. *p < 0.05, v.s. baseline.

With the help of MoCA scale, we compared MoCA score in the rTMS treatment group with that in the sham group. Firstly, our data identified that the MoCA score in the treatment group and sham group showed no significant improvement at each time point. However, we found a significant increase in the mild group at 6 weeks after the end of the treatment compared with the baseline (*p* = 0.046) (Figure 3, Table 2). However, the MoCA score in the moderate treatment group was insignificantly improved compared with that in the moderate sham group (Figure 3, Table 2), indicating that rTMS has more advantages in treating the mild AD patients.

### WHO-UCLA AVLT score

**Figure 4:**
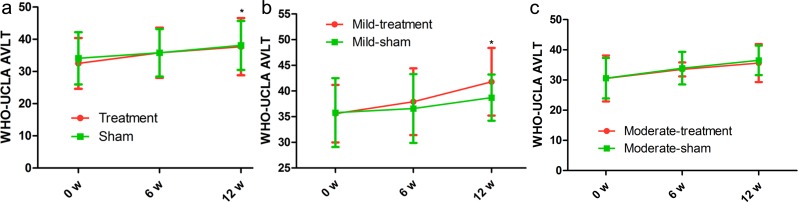
Differences in WHO-UCLA AVLT score at each time point (baseline, immediately after, 6 weeks after the treatment) There was no significant time-by-group interaction in spite of significant improvements in the treatment group. The red line represents the treatment group and the green line represents the sham group. *p < 0.05, v.s. baseline.

Based on precise data of cognitive scores, more cognitive assessment tests were completed in the present study, and we used the WHO-UCLA AVLT score to further assess cognitive level of AD patients. Compared with the baselines, the general level of AVLT in the treatment group has much more improvement by 6.2 points at 6 weeks after the end of the treatment (*p* = 0.039; Table 2). In addition, scores of WHO-UCLA AVLT in the treatment group were significantly higher compared with the baseline level at 6 weeks after the end of the treatment (*p* = 0.021; Table 2); however, scores of WHO-UCLA AVLT in the sham group were no significantly changed compared with the baseline level at 6 weeks after the end of the treatment (*p* = 0.815, *p* = 0.327, respectively; Table 2). Likewise, the AVLT score in the moderate treatment group was also insignificantly improved compared with that in the moderate sham group (Figure 4), indicating that rTMS has more advantages in treating the mild AD patients.

### More findings

In this work, we further assessed cognitive domains, and observed that there was an improvement in the memory and language aspect of cognitive domains when AD patients underwent the rTMS treatment. It should be noted that these findings became more significant and effective in the mild AD patients (Table 3). At the same time, there was no statistically significant improvement in the cognitive level in the moderate AD patients (Table 3), indicating that rTMS may have no effect on moderate AD patients.

**Table 3:** Changes of cognitive domains in the rTMS treatment group

group	cognitive domains	changes of scores, mean (SD)		*p* value		
		Δ immediate after treatment	Δ 6 weeks after treatment	[B] vs. [6]	[B] vs. [12]	time*group
treatment	memory	2.04 (2.25)	2.62 (3.66)	0.002*	0.009*	0.575
	language	1.17 (1.36)	1.39 (1.67)	0.003*	0.003*	0.676
	executive function	0.86 (1.89)	0.49 (1.67)	0.079	0.244	0.549
mild treatment	memory	2.56 (2.33)	2.88 (2.87)	0.003*	0.005*	0.767
	language	1.36 (1.56)	1.54 (1.67)	0.012*	0.009*	0.788
	executive function	0.92 (1.12)	0.92 (1.50)	0.016*	0.058	1.000
moderate treatment	memory	1.56 (2.32)	2.41 (3.12)	0.207	0.159	0.638
	language	0.98 (0.89)	1.15 (1.56)	0.070	0.175	0.838
	executive function	0.74 (0.99)	0.00 (1.11)	0.170	1.000	0.298

Repetitive measures analysis of covariance includes age, gender and education level as covariates; Bonferroni correction is used for multiple comparisons.

*p value<0.05.

Δ: differences from baseline to at each point, [B]: baseline, [6]: immediately after the end of treatment, [12]: 6 weeks after the end of treatment.

## DISCUSSION

Recently, repetitive transcranial magnetic stimulation has been successfully applied in diverse neuropsychiatric disorders and diseases, particularly in AD, PD and some depression symptoms [13, 15, 16]. High-frequency rTMS (more than 5 Hz) has been reported to deliver its signal into left dorsolateral prefrontal cortex [17, 18]. However, low-frequency rTMS (less than 1 Hz) is able to control the activity of the right dorsolateral prefrontal cortex [19, 20]. To date, the clinical role of rTMS in treatment of AD patients with cognitive deficits is still not elucidated and some results are conflicting. In the present study, we enrolled 30 AD patients and conducted the rTMS and non-rTMS therapy. We identified that a significant increase in the cognition level could be found when AD patients underwent rTMS treatment in this trial. Additionally, the neuropsychological assessments including ADAS-cog, MMSE, MoCA and WHO-UCLA AVLT showed a significant improvement in mild AD patients with the treatment of rTMS. These findings indicatied that rTMS has more advantages in treating the mild AD patients.

Till now, the physiological mechanisms underlying the treatment efficacy of rTMS have been not wholly elucidated. Some reports suggested that magnetic field of rTMS has the capacity of directly modulating specific cortical regions as well as neural networks [21]. In addition, because the memory and learning activity can be regulated by synaptic neuronal activities, and synaptic strength can be altered by co-activation of input neurons, rTMS may promote the neural coactivation to change synaptic strength to further facilitate memory and learning activity [21]. In general, high-frequency rTMS can be located into various cortical areas, which was consistent with related cognitive function in this work. Thus, rTMS might enhance the possibility of cortical plasticity, and then transduct magnetic signals to the associated cortical regions. Most reports identified that there were no significant discrepancies in cognitive change between rTMS and sham non-rTMS.

However, other studies also found that rTMS has the beneficial effects on different cognitive level, including memory, language, and executive function. Maria et al identified that rTMS has an important role in improving language and auditory sentence comprehension [22–24], and then another report also showed that rTMS can facilitate verbal and nonverbal agility, and attenuate some cognitive indicators in AD patients [25]. In accordance with these results, our findings showed that the language and memory domains were also obviously improved in AD patients with rTMS, especially in the mild treatment group.

The present study had several limitations: (1) the small sample size (a total of 30 patients) was applied in this study, which limited its statistical power and resulted in lack of actual effects. (2) The effectiveness of double-blinding and random sequence was not well evaluated, leading to bad impact on the validity of clinical trial and statistics. (3) Despite that the average donepezil level did not show any significant differences between the treatment and the sham groups, the dosage of donepezil was not well defined at the beginning of the trial, and the blood detection of donepezil was not conducted in this trial. Thus, the other role of donepezil may influence the actual effect of rTMS on AD patients; (4) the frequency of rTMS might not be enough to obtain effective clinical data, which may need long-time or higher frequency treatment.

In conclusion, this study suggested that repetitive transcranial magnetic stimulation improves cognitive level, memory and language of AD patients, especially in the mild stage of AD patients. Thus, rTMS can be recommended as a promising adjuvant therapy combined with cholinesterase inhibitors at the mild stage of AD patients. Further trials with larger sample size are available to determine the optimal treatment indicators, involving rTMS orientation and periods.

## MATERIALS AND METHODS

### Ethics statement

The ethical committee of Qilu Hospital of Shandong University had approved all procedures and all subjects gave their written informed consent prior to participation. The study was performed in agreement with the Declaration of Helsinki.

### Study population

#### Inclusion criteria

Thirty patients (17 patients receiving rTMS treatment and 13 receiving sham treatment) diagnosed with AD based on the diagnostic criteria of the Diagnostic and Statistical Manual of Mental Disorders, 4th edition were recruited for this study. Their MMSE score was 18-26 and their global Clinical Dementia Rating scale score was 1 or 2. All of the participants in this study were required to be accompanied by a caregiver or a family member who spent more than 72 hours per week with the patient to provide daily information about them. All patients could read and write Chinese proficiently, and brain MRI was performed to exclude any organic brain lesions that might have affected cognitive function. The patients were required to maintain their drugs without changes from at least 2 months before the start of the study and throughout its duration.

#### Exclusion criteria

Patients with a history of alcohol abuse or who had taken psychoactive medications within the past month were excluded. Patients who were not capable of touching a computer screen, who were unable to cooperate with the technician because of vision or hearing difficulty, or who had contraindications for rTMS were also excluded, as were patients who were not available for general TMS treatment.

### Study design

This was a prospective, randomized, double-blind, placebocontrolled study that was conducted from February 2014 to February 2015. The patients were randomly assigned to the treatment or sham-treated group. The patients in the treatment group received daily treatment sessions for 6 weeks (1 session/day and 5 days/week for total of 30 sessions), while the sham-treated group received regular sham management without stimulation. The treatment areas included parietal P3/P4 and posterior temporal T5/T6 according to electroephalogram 10-20 system. Neuropsychological assessments were performed before the treatment and immediately after and 6 weeks after the end of rTMS.

### Treatment procedure

The patients in the treatment group received daily treatment sessions for 6 weeks (1 session/day and 5 days/week for total of 30 sessions in 6 weeks). Each session lasted 1 hour, including preparation, and three brain areas were targeted and stimulated separately. 10 min of rTMS (10 s of 20 Hz/train, 20s intermediate/train) followed by two to four cognitive tasks were administered over the course of 20-40 s for each brain area. For patients in the sham group, the same coil was positioned for stimulating the same selected brain areas but without applying any magnetic stimulation, and the patients heard the same sounds that had been recorded for when stimulation was applied to the other patients. All AD patients were administrated with the same basic treatment such as cholinesterase inhibitors and donepezil.

### Clinical assessments and analyzed variables

All measurements of clinical assessments were repeated three times: at baseline and then immediately and 6 weeks after the end of TMS treatment. The initial evaluation was performed 2 weeks before starting TMS treatment. The second evaluation was performed at the time that the 6-weeks stimulation period was completed, and the final evaluation was performed 6 weeks after the end of TMS treatment. Patients were also divided into two groups according to the severity of AD using the cutoff MMSE score of 21: mild group, 21-26; and moderate group, < 20. All cognitive assessments were performed by a trained neuropsychologist who was blinded to the treatment status of the participants (i.e., treated or sham-treated) throughout the study.

### Neuropsychological tests

Semi-structured interviews were used to collect all subjects’ demographic data, vascular risk factors (hypertension, diabetes, high cholesterol, heart disease, smoking, etc.) as well as their current and past medical histories. All subjects were also given a detailed neurological examination and neuropsychological assessment. The main test questionnaire included:

The Chinese version of Alzheimer's Disease Assessment Scale-cognitive subscale (ADAS-cog). It was measured at each time point. The ADAS-cog, a widely used cognitive assessment instrument in AD clinical trials, consists of memory, language, orientation, and praxis assessments. Its scores range from 0 to 70, with higher values indicating higher degree of deficit.

The Chinese version of Mini Mental State Examination (MMSE). It includes six factors: orientation, immediate recall, attention, short-term memory, and language, with a maximum score of 30.

The Chinese version of Montreal Cognitive Assessment (MoCA). It includes attention and concentration, executive functions, memory, language, visuospatial abilities, abstract thinking, calculating abilities and orientation. The cutoff value of MoCA on Chinese population is ≥ 26 with education ≤ 12. The final score is the actual measured score plus one point.

The Chinese version of World Health Organization University of California-Los Angeles, Auditory Verbal Learning Test (WHO-UCLA AVLT): This test primarily for verbal memory, patients’ short-term memory and the ability to learn new things, including immediate recall, delayed recall, long delayed recognition.

### Statistical analysis

Demographics were analyzed using the Mann-Whitney U test for continuous variables and Fisher's exact test for categorical variables. All of the assessments were analyzed *via* repeated-measures analysis of covariance, including age, gender, and duration of education as covariates to evaluate the consecutive changes in each score from baseline to after rTMS treatment between the treatment and sham groups. The scores obtained immediately and 6 weeks after the end of rTMS-COG treatment were compared with those obtained at baseline by multiple comparisons with Bonferroni correction. All of these statistics were applied identically in the subgroup analysis. All analyses were performed using SPSS software (version 19.0, SPSS Inc., Chicago, IL, USA). Two-sided probability values of *p* < 0.05 were considered statistically significant.
